# Facial Shape Analysis Identifies Valid Cues to Aspects of Physiological Health in Caucasian, Asian, and African Populations

**DOI:** 10.3389/fpsyg.2017.01883

**Published:** 2017-10-30

**Authors:** Ian D. Stephen, Vivian Hiew, Vinet Coetzee, Bernard P. Tiddeman, David I. Perrett

**Affiliations:** ^1^Department of Psychology, Macquarie University, Sydney, NSW, Australia; ^2^ARC Centre of Excellence in Cognition and its Disorders, Macquarie University, Sydney, NSW, Australia; ^3^Perception in Action Research Centre, Macquarie University, Sydney, NSW, Australia; ^4^School of Psychology, University of Nottingham Malaysia Campus, Semenyih, Malaysia; ^5^Department of Genetics, University of Pretoria, Pretoria, South Africa; ^6^Department of Computer Science, Aberystwyth University, Aberystwyth, United Kingdom; ^7^School of Psychology and Neuroscience, University of St. Andrews, St. Andrews, United Kingdom

**Keywords:** face perception, health perception, geometric morphometrics, evolutionary psychology, facial appearance

## Abstract

Facial cues contribute to attractiveness, including shape cues such as symmetry, averageness, and sexual dimorphism. These cues may represent cues to objective aspects of physiological health, thereby conferring an evolutionary advantage to individuals who find them attractive. The link between facial cues and aspects of physiological health is therefore central to evolutionary explanations of attractiveness. Previously, studies linking facial cues to aspects of physiological health have been infrequent, have had mixed results, and have tended to focus on individual facial cues in isolation. Geometric morphometric methodology (GMM) allows a bottom–up approach to identifying shape correlates of aspects of physiological health. Here, we apply GMM to facial shape data, producing models that successfully predict aspects of physiological health in 272 Asian, African, and Caucasian faces – percentage body fat (21.0% of variance explained), body mass index (BMI; 31.9%) and blood pressure (BP; 21.3%). Models successfully predict percentage body fat and blood pressure even when controlling for BMI, suggesting that they are not simply measuring body size. Predicted values of BMI and BP, but not percentage body fat, correlate with health ratings. When asked to manipulate the shape of faces along the physiological health variable axes (as determined by the models), participants reduced predicted BMI, body fat and (marginally) BP, suggesting that facial shape provides a valid cue to aspects of physiological health.

## Introduction

Evolutionary accounts of human facial attractiveness posit that facial cues associated with attractiveness and healthy appearance represent valid cues to aspects of underlying physiological health. Over the last two decades, researchers have successfully identified facial cues that influence facial attractiveness and apparent health (attractiveness and apparent health are closely related; Jones et al., 2004): symmetry (Grammer and Thornhill, 1994), averageness (Rhodes et al., 2001b), sexual dimorphism (Perrett et al., 1998), skin color (Fink et al., 2006; Matts et al., 2007; Stephen et al., 2009b, 2012), facial adiposity (Coetzee et al., 2009), and skin homogeneity (Matts et al., 2007) all being identified as contributing to attractiveness or healthy appearance. In order to identify a valid cue to health, however, it is also necessary to demonstrate a link between the cue in question and some aspect of real, physiological health. This part of the equation has received much less attention (Coetzee et al., 2009).

Facial symmetry has been found to contribute to attractiveness in Western (Grammer and Thornhill, 1994; Perrett et al., 1999; Penton-Voak et al., 2001) and non-Western industrialized (Rhodes et al., 2001a) and traditional (Little et al., 2007) societies, as well as being preferred in opposite sex conspecifics by non-human primates (Waitt and Little, 2006). It has been suggested that low fluctuating asymmetry reflects developmental stability, since individuals whose development is not interrupted by illness and malnutrition can develop more evenly (Grammer and Thornhill, 1994; Swaddle and Cuthill, 1995). Attempts to empirically link facial symmetry to developmental health have been more mixed. Thornhill and Gangestad (2006) found positive associations between both facial and body fluctuating asymmetry and susceptibility to respiratory, but not intestinal infections. In contrast, Rhodes et al. (2001b) found that facial symmetry did not correlate with childhood, adolescent or current health, judged from medical records. A similar study using a large longitudinal database also failed to find a relationship between facial symmetry and health during development (Pound et al., 2014).

Facial averageness is frequently linked to attractiveness (Langlois and Roggman, 1990; Rhodes et al., 2001b; Rhodes and Tremewan, 2010), and is thought to reflect a high degree of heterozygosity in the genome (Lie et al., 2008), as well as a lack of deleterious alleles (Thornhill and Gangestad, 1993). Rhodes et al. (2001b) showed a negative relationship between distinctiveness (the inverse of averageness) and some aspects of actual health, as rated from medical records. Foo et al. (2017) find a positive relationship between distinctiveness and semen quality but no relation of distinctiveness to immune function.

Skin condition is associated with attractiveness and healthy appearance, with skin color (Stephen et al., 2009a,b, 2012; Scott et al., 2010; Coetzee et al., 2012) and skin color distribution (Fink et al., 2006, 2012; Matts et al., 2007; Coetzee et al., 2012) influencing perceptions of health and attractiveness. Further, healthy appearing skin color is associated with aspects of real health such as a diet rich in antioxidant carotenoids (Stephen et al., 2011; Whitehead et al., 2012) and an even skin color distribution is associated with reduced damage by ultraviolet light (Matts and Fink, 2010), suggesting that skin appearance is related to both healthy/attractive appearance and aspects of real health. Further support for this hypothesis comes from the finding that heterozygosity at Major Histocompatability loci is correlated with healthy appearance of male faces and skin patches (Roberts et al., 2005), though Coetzee et al. (2007) and Lie et al. (2008) failed to replicate this finding in women’s faces.

More recently, Coetzee and colleagues demonstrated that facial adiposity (perceived weight, as rated from the face) predicted facial attractiveness in Caucasian (Coetzee et al., 2009; Rantala et al., 2013) and female African populations (Coetzee et al., 2012). Facial adiposity was also significantly associated with perceived health and physiological health measures (BMI, blood pressure and the frequency and severity of cold and flu bouts; Coetzee et al., 2009). Further studies found a significant association between facial adiposity and longevity (Reither et al., 2009), women’s physical and psychological condition (Tinlin et al., 2012), women’s salivary progesterone levels (Tinlin et al., 2012) and a direct measure of men’s immune response (antibody response to Hepatitis B vaccination; Rantala et al., 2013), indicating that facial adiposity is a robust facial cue to health. Coetzee et al. (2010) identified three structural cues associated with facial adiposity across ethnic and sex boundaries: facial perimeter-area ratio, width to height ratio and cheek-to-jaw width, and Wen and Guo (2013) found relationships between BMI and seven facial measures.

Obesity (BMI > 30) and overweight status (BMI > 25) are associated with a range of health problems, particularly cardiovascular health, including hypertension and cardiovascular disease (Hubert et al., 1983; Manson et al., 1995; Lusky et al., 1996; Wilson et al., 2002). BMI is also strongly correlated with percentage body fat (Ranasinghe et al., 2013), but this is not a perfect relationship, since BMI will also be increased in individuals with higher muscle mass or even bone mass (Garn et al., 1986). Percentage body fat is also associated with increased risk of cardiovascular disease (Deurenberg-Yap et al., 2002) and, while the relationship between percentage body fat and fat distribution in the torso is known to vary by age, ethnicity and sex (Deurenberg-Yap et al., 2002), studies have not previously addressed the relationship between percentage body fat and facial shape. Similarly, hypertension (high blood pressure) is associated with increased incidence of stroke and coronary heart disease (MacMahon et al., 1990), but previous work has not assessed the relationship between blood pressure and facial shape.

These previous studies have tended to examine individual facial cues in isolation, and have required that the cues to be studied are identified *a priori*. Further, many of these previously identified cues are interrelated, meaning that investigating each separately can lead to overestimates of their predictive value (Phalane et al., 2017). Now, geometric morphometric methodology (GMM) techniques provide a “bottom–up,” data-driven approach that allow the statistical models to identify important patterns in the data, eliminating the need to identify cues of interest *a priori*, and allowing the examination of multiple facial shape cues simultaneously (Said and Todorov, 2011; Holzleitner and Perrett, 2015; Wolffhechel et al., 2015). GMM is a technique that has been developed to allow landmark-based analysis of shape variation within a population of shapes, to allow the visualization of resultant patterns of variation (Adams et al., 2004). The technique has been applied to analyses of cranial development in primates (O’Higgins and Jones, 1998), geographical variation in cranial shape in humans (Hennessy and Stringer, 2002) and, more recently, measurements of morphological masculinity from human facial photographs (Scott et al., 2010; Stephen et al., 2012), and used to predict reproductive success of humans (Pflüger et al., 2012) and, more recently, BMI (Wolffhechel et al., 2015) and men’s upper body strength (Holzleitner and Perrett, 2015). In the current study, we use GMM to build models to predict aspects of underlying physiological health – BMI, body fat percentage and blood pressure – and rated apparent health from facial shape. In this way, we produce models that predict risk factors of heart disease from face shape. If facial shape represents a valid cue to health, models produced by using facial shape data to predict health variables should also predict rated health, while models produced to predict health ratings based on shape should also predict measured physiological health variables. In a second study, we use the models produced in Study 1 to manipulate the predicted BMI, blood pressure and body fat (separately) of faces, and ask observers to make the faces as healthy as possible. If our models describe valid facial shape cues to health, participants are expected to decrease predicted BMI, blood pressure and body fat to enhance healthy appearance.

## Study 1

### Methods

All work was approved by the relevant ethics committees at the University of Nottingham Malaysia Campus, University of St. Andrews and University of Pretoria as appropriate.

### Photography

One hundred Malaysian Chinese (50 male), 75 United Kingdom-based Caucasian (35 male) and 97 black South African (50 male) participants were photographed (in 2D) in a booth painted with Munsell N5 standard gray paint, and illuminated by daylight simulation tubes (Verivide, United Kingdom). Participants wore headbands to hold hair back from the face, were face on to the camera and were asked to maintain a neutral expression. None of the participants had any visible facial deformity.

### Physiological Measurements

Systolic and diastolic blood pressures were measured using a portable blood pressure monitor. Principal components analysis (PCA) revealed a single underlying component with eigenvalue > 1, which explained 76.76% of variance in the two blood pressure parameters. Participants were asked to remove shoes and socks and all heavy items from their pockets, and were measured for height and weight, and BMI was calculated as weight/height^2^. African and Asian participants were also measured for percentage body fat using a Tanita SC330S body composition analyzer (Tanita, Netherlands).

### Health Ratings

Twenty Malaysian Chinese participants (10 male, 10 female; aged 18–24) at the University of Nottingham Malaysia Campus rated the apparent health of the Malaysian Chinese face photographs. African male faces were rated for apparent health by 15 female and 15 male black African participants (aged 18–30), and African female faces were rated for apparent health by 16 female and 14 male black African participants (aged 18–30) at the University of Pretoria, South Africa. The Caucasian faces were rated by 19 male and 29 female Caucasian participants (aged 18–32) at the University of St Andrews, United Kingdom. All faces were rated on a seven-point Likert-type scale (0 = very unhealthy to 6 = very healthy). Descriptive statistics of physiological and ratings data are presented in **Table 1**.

**Table 1:** Descriptive statistics for physiological health variables and rated health.

Ethnicity	Sex	Measurement	*n*	Mean	*SD*	Minimum	Maximum	Q1	Median	Q3
		BMI	50	19.82	3.47	14.00	36.10	17.70	19.50	21.15
		Percentage body fat	50	23.98	6.80	10.10	48.10	18.58	23.25	28.53
	Female	Blood pressure (systolic)	50	110.08	12.24	82.00	140.00	101.00	109.50	119.00
		Blood pressure (diastolic)	50	68.90	8.09	54.00	85.00	62.00	68.50	75.00
Asian		Rated health	50	4.57	0.52	3.60	5.80	4.10	4.58	4.90
		BMI	50	21.04	3.61	15.50	33.40	18.20	20.35	23.45
		Percentage body fat	50	14.13	6.04	3.20	31.20	9.40	13.65	18.63
	Male	Blood pressure (systolic)	50	118.00	13.54	93.00	153.00	108.75	116.50	129.00
		Blood pressure (diastolic)	50	69.30	7.43	53.00	91.00	64.00	69.50	73.25
		Rated health	50	4.31	0.75	2.60	5.30	3.84	4.50	5.00
		BMI	52	24.27	6.14	17.40	42.90	20.05	21.60	28.20
		Percentage body fat	52	28.90	9.85	9.80	50.30	21.43	27.85	37.03
	Female	Blood pressure (systolic)	52	112.28	8.99	93.50	134.50	106.00	111.25	118.00
		Blood pressure (diastolic)	52	72.83	6.73	58.00	90.50	69.00	72.75	78.00
African		Rated health	49	3.80	0.55	2.81	5.07	3.38	3.78	4.15
		BMI	48	21.14	2.97	15.90	28.10	18.90	20.70	22.70
		Percentage body fat	48	12.19	5.36	3.00	26.30	7.95	12.05	15.60
	Male	Blood pressure (systolic)	49	122.71	12.37	97.50	153.50	115.25	121.00	130.25
		Blood pressure (diastolic)	49	71.91	9.12	55.00	97.50	65.75	72.00	77.25
		Rated health	47	3.78	0.59	2.42	5.29	3.32	3.87	4.16
		BMI	53	22.66	3.25	17.82	31.51	20.85	22.34	25.15
		Percentage body fat	0	–	–	–	–	–	–	–
	Female	Blood pressure (systolic)	53	115.59	10.04	92.00	141.00	109.75	115.50	121.50
		Blood pressure (diastolic)	53	68.80	6.59	53.00	85.00	64.50	69.50	73.00
Caucasian		Rated health	43	3.17	0.77	1.64	4.77	2.57	3.17	3.85
		BMI	46	23.28	2.75	18.42	33.38	21.65	23.11	23.98
		Percentage body fat	0	–	–	–	–	–	–	–
	Male	Blood pressure (systolic)	45	126.74	11.44	104.00	155.00	117.50	127.50	135.00
		Blood pressure (diastolic)	45	69.26	8.44	53.50	93.00	63.75	68.50	76.00
		Rated health	41	3.00	0.84	1.69	4.88	2.34	2.95	3.64

*Untransformed data.*

### Modeling

**FIGURE 1:**
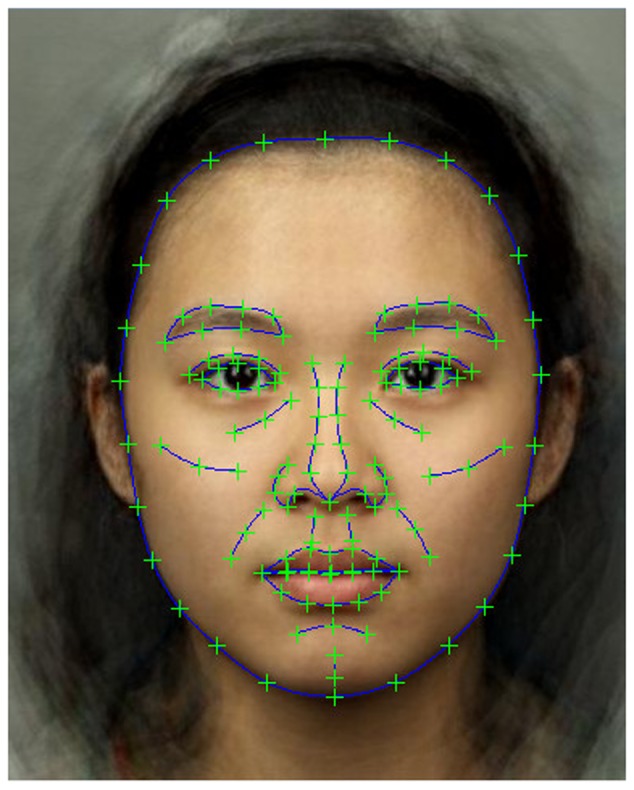
Locations of the 138 delineated landmark points. A composite face is shown for illustrative purposes. Faces used in both modeling and rating parts of the study were real individuals.

Geometric morphometric methodology was used to analyze the shape variation in the sample of facial photographs (O’Higgins and Jones, 1998). Photographs were delineated with 138 landmarks using Psychomorph software (Tiddeman et al., 2001; **Figure 1**). GMM modeling was then performed on all faces together (all three ethnicities and both sexes) using Morphologika 2.5 software (O’Higgins and Jones, 1998), landmarks were subjected to Procrustes registration to remove rotational, scale and translational differences from the individual faces. PCA was then performed on Procrustes-registered landmark data using Morphologika to identify the underlying dimensions of variation in landmark data. Kaiser’s criterion was used to retain 28 orthogonal components, which together accounted for 91.05% of the variance.

The BMI variable was transformed to normality using an inverse transformation, and the percentage body fat variable was transformed to normality using a square root transformation.

Hierarchical linear regression was used to produce models to predict the dependent variables (percentage body fat, BMI, blood pressure factor, and rated health) from facial shape components. Since women were found to have a significantly higher percentage body fat [*t*(198) = 13.203; *p* < 0.001] and lower blood pressure factor [*t*(297) = -3.913; *p* < 0.001] than men, sex was included as a dummy variable in these analyses. No sex difference was found in BMI [*t*(297) = 0.753; *p* = 0.452] or rated health [*t*(278) = 1.215; *p* = 0.225]. A significant difference was found between the different ethnic groups for BMI (*F*_2,298_ = 18.840; *p* < 0.001; Asians had lower BMI than Caucasians or Africans, both *p* < 0.001 but no significant difference between Caucasians and Africans, *p* > 0.05), blood pressure factor (*F*_2,298_ = 4.061; *p* = 0.018; Asians had lower blood pressure than Africans, *p* = 0.018, all other comparisons *p* > 0.05) and rated health (*F*_2,279_ = 93.769; *p* < 0.001; Asians were rated healthier than Africans and Caucasians, Africans were rated healthier than Caucasians, all *p* < 0.001) — but not percentage body fat [*t*(181.568) = 0.790; *p* = 0.431] — so ethnicity was included as a pair of dummy variables (African and Asian) for the BMI, blood pressure and rated health analyses. The initial step in the hierarchical model included sex and ethnicity variables (full dummy coded) as described above. The second step in the hierarchical model added the Principal components (PCs) to the model. Initially, the African dummy variable was found to have high (>10.2) variance inflation factor (VIF) values in the second step of the regression models for BMI, blood pressure and rated health analyses, indicating that multicollinearity between the African dummy variable and one or more of the PCs was a problem, due to one or more PCs describing the shape difference between African and Caucasian (as the comparison group in the dummy coding) faces. Therefore, a linear regression was run to identify the PC to be excluded (Dependent Variable = African, Independent Variables = the 28 PC variables; Asian faces excluded). PC2 was found to strongly predict the African dummy variable, and so was removed from the regression models predicting BMI, blood pressure and rated health. All VIF values were then within the acceptable range (all mean VIFs < 2).

For each model, leave one out cross-validation (LOOCV; n-fold cross-validation) was performed. For each model, DfFit scores were saved, and subtracted from the predicted values. This is equivalent to producing a model from all but one of the cases, then using this model to predict the value of the “left out” case. This is repeated for all possible “left out” cases. Pearson’s r was then used to compare these LOOCV values with the predicted values from the model, allowing us to assess the generalizability of the model. Mean squared error (MSE) values are also reported between cross-validated and model values.

Predicted values and LOOCV predicted values of the physiological measurements were saved and Pearson’s r was used to assess the relationships between these values from the models and rated health.

Finally, since it is known that individuals with higher BMI and percentage body fat are likely to have higher blood pressure, we used hierarchical linear regression analysis to determine whether facial shape could predict blood pressure more effectively than BMI and percentage body fat. Due to the high degree of multicollinearity between BMI and percentage body fat (VIF > 14), two separate analyses were performed, one for BMI and one for percentage body fat. For each, blood pressure was the dependent variable. Model one contained only the sex and ethnicity dummy variables. Model two added BMI or percentage body fat, and model three added the PCs.

### Results

For the BMI hierarchical regression analysis, the first model, which included only the ethnicity dummy variables, explained 11.4% of the variance (*R*^2^= 0.114; *F*_2,267_ = 17.113; *p* < 0.001). The second model, which also included the face shape PCs, explained 43.3% of the variance in BMI (*R*^2^= 0.433; *F*_29,240_= 6.317; *p* < 0.001), 31.9 percentage points more variance in BMI than the first model (*R*^2^_change_= 0.319; *F*_change27,240_ = 5.004; *p* < 0.001). All VIF values were in the acceptable range (mean VIF = 1.349). LOOCV values were highly correlated with predicted values [*r*(270) = 0.986; *p* < 0.001] and MSE was low (<0.01), indicating good generalizability of the model.

For the percentage body fat analysis, the first model, which included only sex, explained 46.3% of variance (*R*^2^= 0.463; *F*_1,194_ = 167.589; *p* < 0.001). The second model, which also included the face shape variables, explained 67.4% of variance (*R*^2^= 0.674; *F*_29,166_= 11.827; *p* < 0.001), 21.0 percentage points more variance than the first (*R*^2^_change_= 0.210; *F*_change28,166_ = 3.824; *p* < 0.001). All VIF values were within the acceptable range (mean VIF = 1.722). LOOCV values were highly correlated with predicted values [*r*(196) = 0.986; *p* < 0.001] and MSE was low (0.02), indicating good generalizability of the model.

For the blood pressure analysis, the first model, which included sex and ethnicity variables, explained 7.3% of the variance (*R*^2^= 0.073; *F*_3,267_= 6.981; *p* < 0.001). The second model, which also included the face shape variables, explained 28.6% of variance (*R*^2^= 0.286; *F*_30,240_= 3.199; *p* < 0.001), 21.3 percentage points more variance than the first (*R*^2^_change_= 0.213; *F*_change27,240_= 2.650; *p* < 0.001). All VIF values were within the acceptable range (mean VIF = 1.446). LOOCV values were highly correlated with predicted values [*r*(271) = 0.974; *p* < 0.001] and MSE was low (0.29), indicating good generalizability of the model.

For the rated health analysis, the first model, which included ethnicity variables, explained 40.4% of variance (*R*^2^= 0.404; *F*_2,268_= 90.778; *p* < 0.001). The second model, which also included the face shape variables, explained 49.2% of variance (*R*^2^= 0.492; *F*_29,241_= 8.044; *p* < 0.001), 8.8 percentage points more variance than the first (*R*^2^_change_= 0.088; *F*_change27,241_= 1.546; *p* = 0.047). All VIF values were within the acceptable range (mean VIF = 1.346). LOOCV values were highly correlated with predicted values [*r*(271) = 0.989; *p* < 0.001] and MSE was low (<0.01), indicating good generalizability of the model.

For the analysis predicting blood pressure, controlling for BMI, the first model, which included only sex and ethnicity dummy variables, explained 40.4% of variance (*R*^2^= 0.404; *F*_3,265_= 59.899; *p* < 0.001). The second model, which included BMI, explained 41.0% of the variance, which was a non-significant increase in explanatory power over the first model (*R*^2^_change_= 0.006; *F*_change1,264_= 2.596; *p* = 0.108). The third model, which included the PCs explained 50.1% of variance in blood pressure, an increase of 9.1 percentage points over the second model (*R*^2^_change_= 0.091; *F*_change27,237_= 1.595; *p* = 0.036). All VIF values were within the acceptable range (mean VIF = 1.486).

For the analysis predicting blood pressure, controlling for percentage body fat, the first model, which included only sex and ethnicity dummy variables, explained 23.0% of variance in blood pressure (*R*^2^= 0.230; *F*_2,192_= 28.640; *p* < 0.001). The second model, which included percentage body fat, predicted 23.7% of variance in blood pressure, a non-significant increase in predictive power (*R*^2^_change_ = 0.008; *F*_change1,191_ = 1.915; *p* = 0.168). The third model, which included the PCs, explained 38.0% of variance in blood pressure, a non-significant increase in predictive power over the second model (*R*^2^_change_ = 0.143; *F*_change27,164_ = 1.403; *p* = 0.103). All VIF values were within the acceptable range (mean VIF = 1.777).

Predicted scores for each health variable model were saved. Thus, a “BMI reflected in facial shape” score was produced, and so on for each health variable. LOOCV cross-validated predicted scores were also produced for each health variable. The predicted and LOOCV predicted scores for rated health correlated significantly with the predicted scores for BMI [*r*(269) = 0.403; *p* < 0.001; LOOCV *r*(269) = 0.401; *p* < 0.001] and blood pressure [*r*(270) = -0.288; *p* < 0.001; LOOCV *r*(270) = -0.285; *p* < 0.001], but not for percentage body fat [*r*(195) = 0.003; *p* = 0.969; LOOCV *r*(195) = 0.016; *p* = 0.829].

**FIGURE 2:**
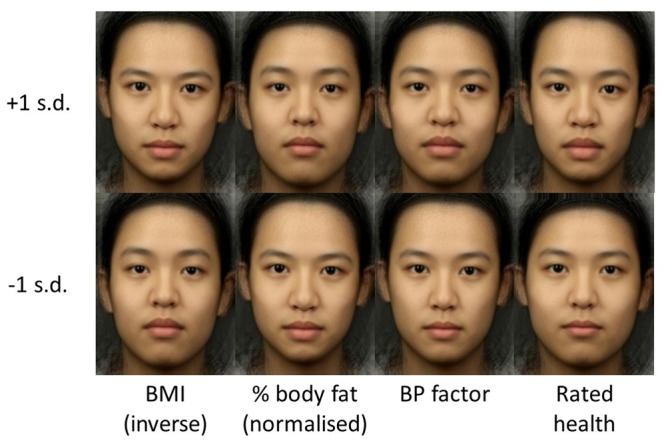
Visualization of linear regression equations: top row is +1 SD and bottom row is -1 SD. Columns correspond (from left to right) to: inverse BMI, normalized percentage fat, blood pressure factor, and health rating.

Using the method of Lagrange multipliers, it is straightforward to show that the optimal shape lies along the vector given by the linear equation parameters scaled by the variance, i.e.,:

$$\begin{array}{c} X_{i}\;=\;\alpha v_{i}n_{i} \end{array}$$

where x_i_ is the ith shape component, n_i_ is the weighting of the ith shape component in the linear model and v_i_ is the variance of the ith shape component. The parameter α can be varied to give a specified value of the dependent variable or a desired probability according to the PCA model. This technique was used to create visualizations of the linear regression equations (**Figure 2**; for visualizations applied to composite faces of each sex and race, see supplementary figures). Values for the sex and ethnicity predictor variables were excluded from this process to ensure that that they were not represented in the visualizations. These visualizations show the most likely facial shape for ±1 SD of the predicted variable according to the PCA shape model.

## Study 2

In order to further investigate the link between our facial shape models of physiological health and perceived health, a perceptual study was conducted in which participants manipulated facial photographs along the BMI, blood pressure and body fat shape dimensions determined by our models, in order to make them appear as healthy as possible.

### Participants

Twenty six Caucasian participants (6 male, 20 female, aged 18–35) were recruited from Macquarie University. Participants received course credit for their time.

### Stimuli

The 138 landmark points were calculated using the linear regression equation for BMI to represent ±1 SD of predicted BMI, as described in supplementary material. These landmark points were loaded into Psychomorph and used as endpoints in a shape transform. Each of 60 faces (10 male and 10 female each from African, Asian, and Caucasian samples; drawn at random from the set used in Study 1) was manipulated by the difference in shape between the two endpoints in 13 steps. For each face, this produced a series of 13 frames (numbered 0–12) in which frame 0 was reduced by 2 SD predicted BMI, increasing incrementally so that frame 7 was the original image and frame 12 was increased by 2 SD predicted BMI. This process was repeated for predicted blood pressure and predicted body fat. A total of 180 trials were produced (2 sexes × 3 ethnicities × 10 identities × 3 manipulations).

### Procedure

Participants were presented with the stimuli, one identity at a time, in a “slider” app. By cycling through the 13 frames, this app allowed participants to manipulate the face along a single model axis (BMI, blood pressure, or body fat) by moving the mouse left and right across the screen. Participants were asked to “make the face as healthy as possible” before clicking the mouse to save the data and move onto the next trial. Trials were blocked by manipulation type (BMI, blood pressure, or body fat), order of presentation was randomized within blocks, and order of blocks was randomized. Location of the transform midpoint on the screen was randomized and presentation was looped to obscure the location of the transform midpoint.

The mean amount of change chosen to enhance healthy appearance (in predicted SD) was saved for each trial type for each participant. One-sample *t*-tests were used to test for significant changes from the original image.

### Results

**FIGURE 3:**
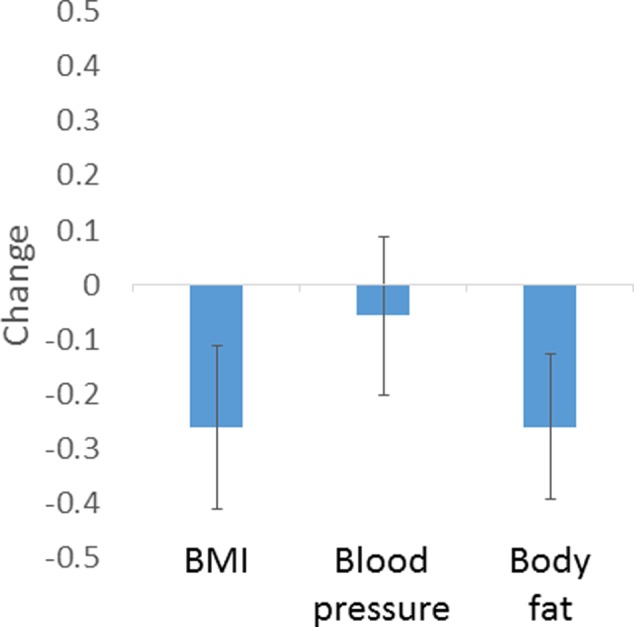
Amount of manipulation (in SD of predicted values) based on regression models chosen by participants to enhance healthy appearance. Error bars show standard error of the mean.

One-sample *t*-tests showed that participants significantly decreased the predicted BMI (mean difference = 0.52 SD, *t* = 8.90, *p* < 0.001) and the predicted body fat (mean difference = 0.51 SD, *t* = 9.91, *p* < 0.001) of faces to enhance their apparent health. There was a non-significant trend in the direction of participants decreasing the predicted blood pressure (mean difference = 0.11 SD, *t* = 1.95, *p* = 0.06; **Figure 3**).

## Discussion

While previous attempts to link facial appearance to aspects of real, underlying physiological health have had mixed results (Thornhill and Gangestad, 1993; Rhodes et al., 2001b, 2003; Pound et al., 2014), the current study has successfully used geometric morphometric modeling (O’Higgins and Jones, 1998) of facial landmark data to predict aspects of underlying physiology that are risk factors for cardiac illness – BMI (32% of variance explained), percentage body fat (21%), and blood pressure (21%). A further model significantly predicted ratings of apparent health. Further, the values of the BMI and blood pressure, but not percentage body fat, as predicted by the regression models, were significantly correlated with rated apparent health. This suggests that the shape cues that vary with aspects of physiological health, blood pressure and BMI, are used by observers in assessing health from people’s faces. This therefore provides support for the hypothesis that the human face contains valid cues to physiological health, and that facial appearance therefore provides a reliable mechanism for identifying healthy and unhealthy individuals.

By using geometric morphometric methods, we avoided the problem of needing to specify facial measurements *a priori*, and instead allowed the statistical model to identify important patterns in the data (Said and Todorov, 2011; Holzleitner and Perrett, 2015). Of course, this “bottom–up,” data-driven approach may in part be capturing variation in previously identified cues to body size, such as perimeter to area ratio, facial width to height ratio, or cheek-to-jaw width ratio (Coetzee et al., 2010), though the *R*^2^ values found for our models explain more variance (*R*^2^_change_ ≥ 0.21) than the individual shape correlates of BMI described in previous studies (*R*^2^ = 0.051 to 0.088; Coetzee et al., 2010). Further, since many previously identified facial cues to health are interrelated, examining each cue in isolation risks overestimating the predictive value of each. In contrast, the bottom–up approach we take in the current study allows the assessment of shape in a more holistic way, and the LOOCV provides confidence that models are not over-fitted. The methods described in this paper provide the tools for bottom–up identification of shape correlates of physiological health, fertility (Peters et al., 2008), and even psychological variables (Boothroyd et al., 2008) from faces and bodies in the future (Holzleitner and Perrett, 2015).

It is interesting to note the differences in physiological measurements and health ratings between the different sexes and ethnic groups. Women are known to have higher body fat than men, probably to facilitate the proper function of the reproductive system through production, metabolism, storage, and binding of estrogen (Frisch, 1987). In line with previous research, women in our sample had lower blood pressure than men, a phenomenon that may be due to smaller stroke volume and lower peripheral resistance in women (Syme et al., 2009). No sex difference was found in BMI or health rating. Ethnic differences were found in BMI, with Asian participants having significantly lower BMI than African or Caucasian participants. This is in line with the finding that East Asian individuals are at increased risk of adverse health outcomes (WHO, 2004), and have higher percentage body fat (Carpenter et al., 2013) than individuals of other ethnicities of similar BMI. African participants had higher blood pressure than Asian participants, again in line with findings that Africans have higher blood pressure than other ethnic groups (Jones and Hall, 2006), though it should be noted that the majority of comparison studies have been conducted in African American populations, whereas the population in the current study was from South Africa. Further, rated health differed between ethnic groups, with Asian participants rated as the healthiest looking, followed by African participants and Caucasian participants rated as least healthy. Since each ethnic group’s faces were rated only by own-ethnicity raters, it may be that there were differences in healthy appearance, or simply that Asian raters have a tendency to give higher scores for the same level of healthy appearance, followed by African and finally Caucasian raters. By including the ethnicity and sex variables in the first step of the hierarchical regression, these potentially confounding factors have been removed from the models reported here. While it is not possible to know the body composition, BMI or blood pressure of ancestral populations, studies have shown that extant hunter-gatherers have BMI, body fat, and metabolisms equivalent to the low end of the healthy range seen in Western societies (Pontzer et al., 2012), suggesting that similar models may be applicable in traditional societies. Future studies should address this question empirically.

We also find that our model using facial shape predicts variance in blood pressure over and above that explained by BMI, ethnicity, and sex information alone. Face shape did not explain additional variance in blood pressure over and above that explained by sex, ethnicity, and percentage body fat – though it should be noted that statistical power was lower in the percentage body fat analysis, due to these data not being known for our Caucasian sample. This suggests that our model using face structure may be a more effective way of predicting health outcomes associated with hypertension than simply measuring BMI.

Finally, we find that participants chose to decrease predicted BMI, body fat and (marginally) blood pressure (based on our models) in order to enhance the healthy appearance of faces. This provides further support for the argument that our models describe valid facial shape-based cues to physiological health. It should be noted that, while the close concordance between perceived attractiveness and perceived health of faces is well-established (Jones et al., 2004), here we examine the relationship between facial shape, healthy appearance and underlying objective aspects of physiological health. Since there have been suggestions that observers overestimate the health of attractive people (Kalick et al., 1998), future studies should establish whether GMM-based models that predict aspects of underlying physiological health are also predictive of facial attractiveness.

## Conclusion

In conclusion then, physiological measures relevant to cardiovascular health are reflected in the shape of the face. By using GMM methodology, models were produced to predict these physiological variables based on facial shape data. The shape variation that predicts the physiological variables also predicts the apparent health of faces, as judged by raters, and enhances the healthy appearance of faces in interactive trials, indicating that the facial shape cues that reflect physiological health are also used by observers to make health judgments. This provides strong support for the hypothesis that the face contains valid, perceptible cues to physiological health.

## Ethics Statement

All work was approved by the relevant ethics committees at the University of Nottingham Malaysia Campus, University of St. Andrews, University of Pretoria and Macquarie University, as appropriate. All participants gave prior, informed consent in writing.

## Author Contributions

Conceived and designed studies: IS, DP, VC, and VH; stimuli production: IS, VH, VC, DP, and BT; data collection: IS and VH; data analysis: IS, VH, and BT; writing and approving manuscript: IS, DP, VC, VH, and BT.

## Conflict of Interest Statement

The authors declare that the research was conducted in the absence of any commercial or financial relationships that could be construed as a potential conflict of interest.
