# Supplementary material for: Facial Shape Analysis Identifies Valid Cues to Aspects of Physiological Health in Caucasian, Asian, and African Populations
Source: Front Psychol. 2017 Oct 30;8:1883. doi: 10.3389/fpsyg.2017.01883 (PMC5670498; doi:10.3389/fpsyg.2017.01883)
Supplement: Supplementary file 1 [file Image_1.PDF]

Supplementary Material

Table S1: structure of the regression models predicting BMI, percentage body fat, blood pressure and rated health (model 2). *p<.05, **p<.01, ***p<.001.

	BMI (inverse transformed)			Percentage body fat (square root transformed)			BP factor			Health rating		
Variable	β	t	p	β	t	p	β	t	p	β	t	p
Gender				-.780	-11.615	<.001***	.149	1.731	.085			
African	.053	.646	.519				.226	2.457	.015*	.363	4.733	.030*
Asian	.463	4.005	<.001***				.074	.567	.571	.668	6.138	<.001***
PC1	-.212	-3.898	<.001***	.099	1.515	.132	.123	2.023	.044*	-.040	-.770	.431
PC2				.056	.688	.492						
PC3	-.035	-.529	.598	.083	.969	.334	.142	1.644	.102	-.065	-1.024	.383
PC4	.242	4.924	<.001***	-.164	-3.199	.002**	-.190	-3.403	.001**	.005	.112	.945
PC5	.195	3.517	.001**	-.078	-1.570	.118	-.071	-1.138	.256	-.065	-1.237	.381
PC6	.037	.736	.462	-.002	-.032	.974	-.016	-.260	.795	.072	1.531	.127
PC7	-.012	-.242	.809	-.031	-.522	.603	.133	2.298	.022*	-.068	-1.395	.155
PC8	-.110	-1.765	.079	.109	1.602	.111	-.014	-.195	.846	-.037	-.632	.533
PC9	.134	2.751	.006**	-.094	-1.570	.118	-.069	-1.156	.249	.041	.885	.414
PC10	-.094	-1.776	.077	-.005	-.087	.931	.215	3.508	.001**	.053	1.064	.406
PC11	.010	.192	.848	-.020	-.406	.685	.013	.220	.826	.070	1.436	.157
PC12	.144	2.866	.005**	-.135	-2.202	.029	-.105	-1.799	.073	.051	1.066	.356
PC13	-.111	-2.086	.038	.086	1.192	.235	-.050	-.806	.421	.005	.107	.883
PC14	.206	3.570	<.001***	-.202	-3.635	<.001***	.088	1.332	.184	-.042	-.763	.413
PC15	.072	1.480	.140	-.080	-1.243	.216	-.084	-1.545	.124	.084	1.820	.068
PC16	-.098	-1.982	.049*	.028	.435	.664	.011	.199	.843	-.060	-1.284	.190
PC17	.024	.499	.619	.021	.446	.656	.050	.901	.368	-.014	-.300	.740
PC18	-.065	-1.315	.190	.057	1.228	.221	.102	1.850	.065	-.072	-1.554	.120
PC19	-.016	-.319	.750	.026	.534	.594	-.123	-2.143	.033*	.013	.287	.774
PC20	.101	2.058	.041*	-.078	-1.599	.112	-.042	-.766	.444	-.038	-.818	.437
PC21	.069	1.376	.170	-.132	-2.412	.017*	-.031	-.558	.578	-.029	-.620	.523

PC22	.097	1.960	.051	-.097	-1.877	.062	-.103	-1.855	.065	.101	2.158	.034*
PC23	.056	1.112	.267	-.037	-.783	.435	-.005	-.086	.932	.143	2.997	.003**
PC24	-.095	-1.894	.059	.084	1.791	.075	.025	.438	.662	-.044	-.928	.349
PC25	.011	.228	.820	.013	.270	.788	-.058	-1.065	.288	-.010	-.213	.850
PC26	.071	1.449	.149	-.023	-.479	.633	-.110	-1.978	.049*	.080	1.738	.087
PC27	.026	.529	.597	-.002	-.040	.968	-.041	-.730	.466	-.050	-1.094	.264
PC28	.112	2.296	.023*	-.105	-2.081	.039*	-.163	-2.970	.003**	.009	.192	.850

Supplementary material: Visualisation of linear regressions

In order to visualise the linear equations derived from regression of the principal component scores (and other factors, such as sex and ethnicity) against the desired prediction variables (inverse transformed BMI, normalised percentage fat, blood pressure and health rating) we can approach it as an optimisation problem. We wish to maximize:

$$\sum_{i=0}^{N} n_i x_i + \alpha$$

subject to the constraint that the probability of the shape is fixed, i.e.

$$\sum_{i=0}^{N} \frac{x_i^2}{v_i} = k$$

where n_i are the coefficients of the linear equation (e.g. for BMI), x_i are the PCA weights, α is a constant (which depends on the linear equation constant, and any other variables included in the model such as sex or ethnicity, which we are not maximizing over, v_i is the variance of the ith principal component and k is the desired negative log probability. To solve this we can use the method of Lagrange multipliers, combining the two equations into a single one:

$$\chi(x_i, \lambda) = \sum_{i=0}^{N} n_i x_i + \alpha + \lambda\left(\sum_{i=0}^{N} \frac{x_i^2}{v_i} - k\right)$$

Differentiating this with respect to λ just gives us back the original constraint equation (as is usual with Lagrange multipliers). Differentiating with respect to a particular x_i and setting the result to zero gives:

$$n_i = -2\lambda \frac{x_i}{v_i}$$

or rearranging gives:

$$x_i = \frac{1}{-2\lambda} v_i n_i$$

This is the equation of a line through the origin. Hence the optimal shape components to give the maximum value for the regression equation (e.g. BMI) at any given shape probability is proportional to the linear equation coefficients scaled by the ***variance*** of each component. We insert these equations back into the linear equation to get a formula for λ in terms of the desired output (e.g. ±1 s.d. of the DV), assuming that the value of α is such that the mean shape gives the mean value for the variable. This implies:

$$\rho = \frac{\sigma}{\sum_{i=0}^{N} v_i n_i}$$

where $\rho = -1/2\lambda$ and σ is the desired standard deviation.

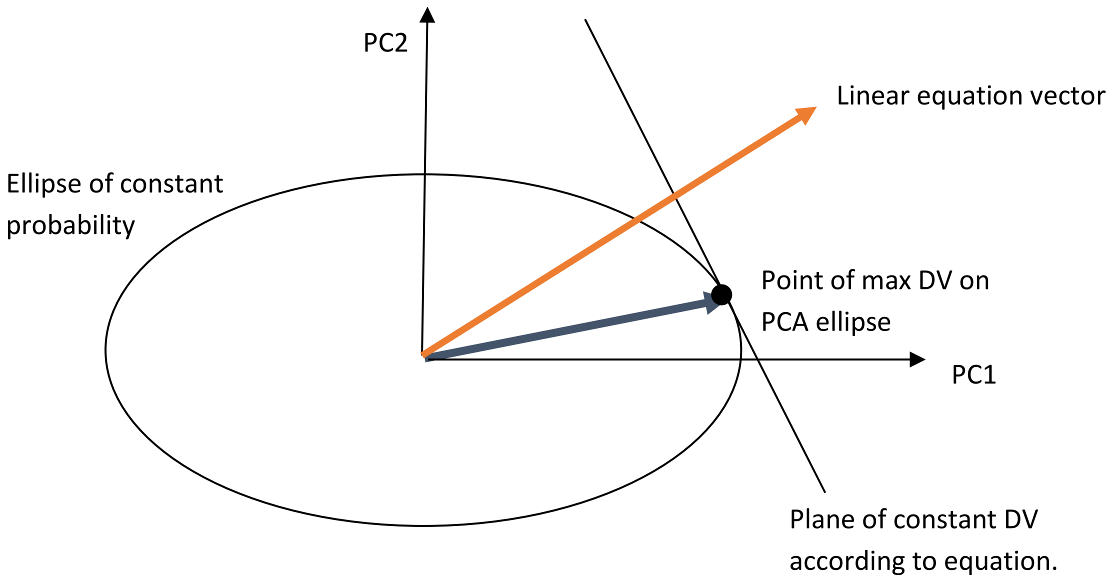

Supplementary figures: Visualisation of linear regression equations applied to composites of each race and sex.

For each race and sex, the top row is +1 s.d. and bottom row is -1 s.d. Columns correspond (from left to right) to: inverse BMI, normalised percentage fat, blood pressure factor and health rating.

Panel 1: Caucasian female

+1 sd

-1 sd

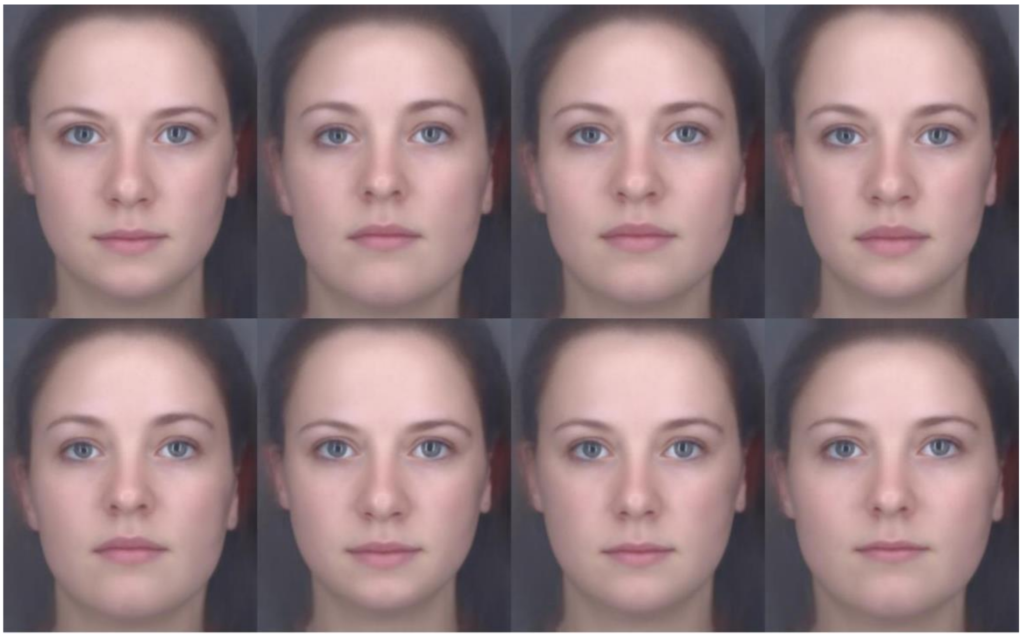

BMI (inverse) | % body fat (normalised) | BP factor | Rated health

Panel 2: Caucasian male

+1 sd

-1 sd

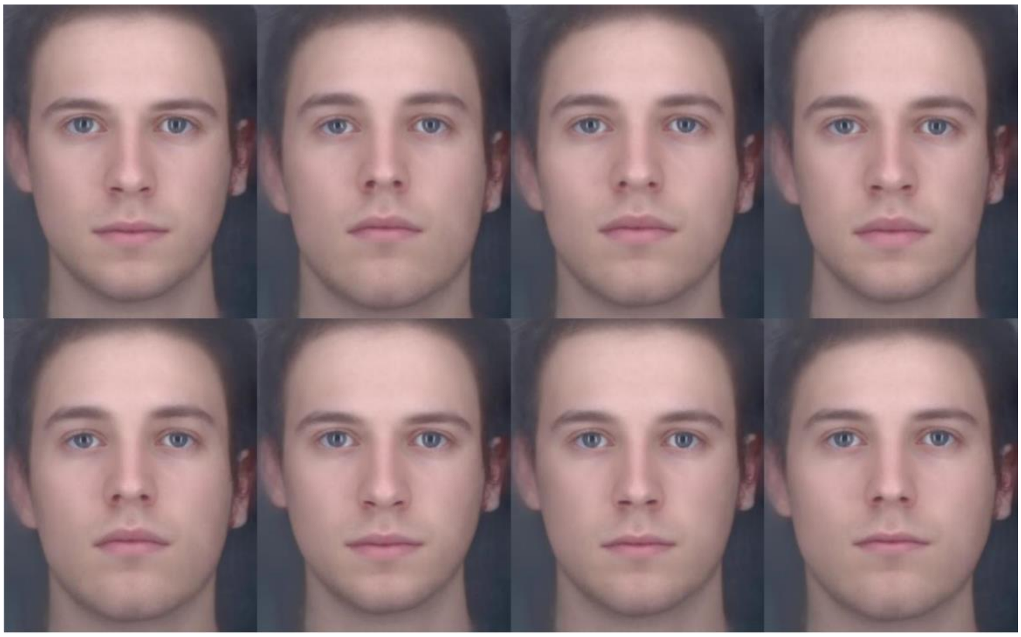

BMI (inverse) | % body fat (normalised) | BP factor | Rated health

Panel 3: African female

+1 sd

-1 sd

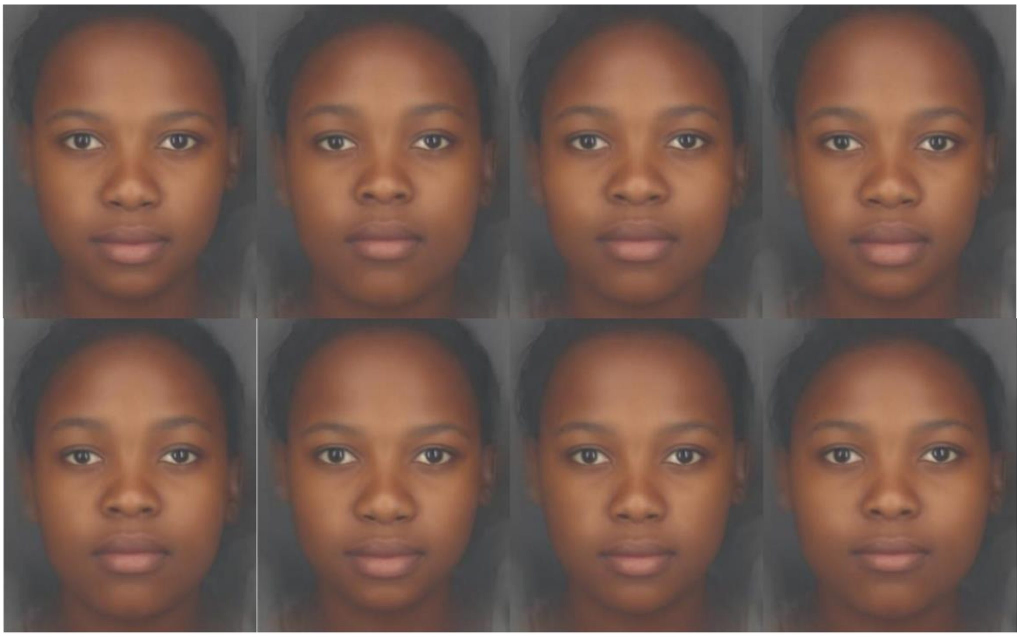

BMI (inverse) | % body fat (normalised) | BP factor | Rated health

Panel 4: African male

+1 sd

-1 sd

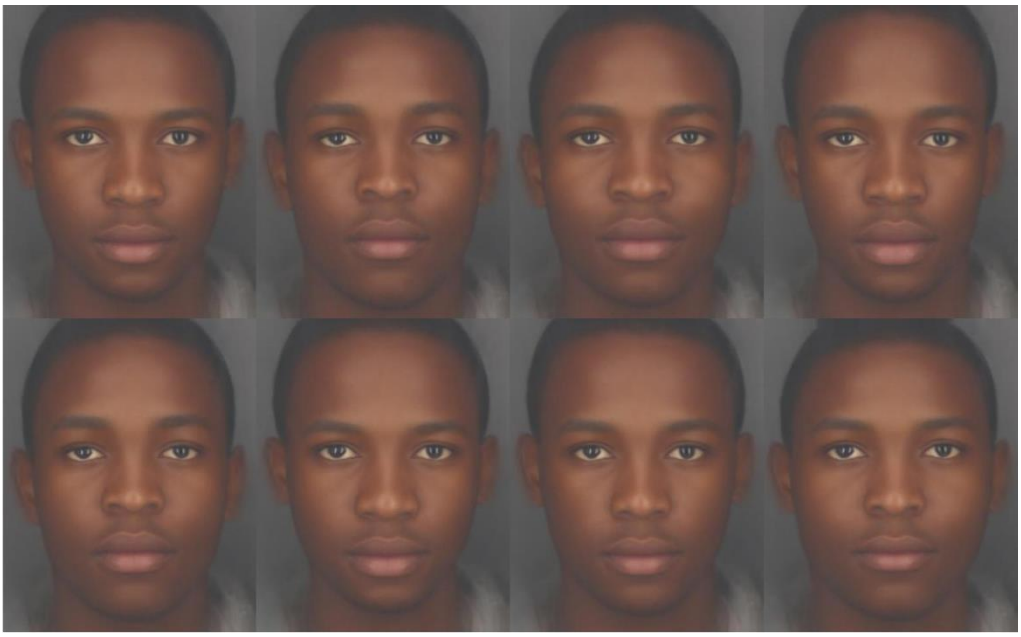

BMI (inverse) | % body fat (normalised) | BP factor | Rated health

Panel 5: Asian female

+1 sd

-1 sd

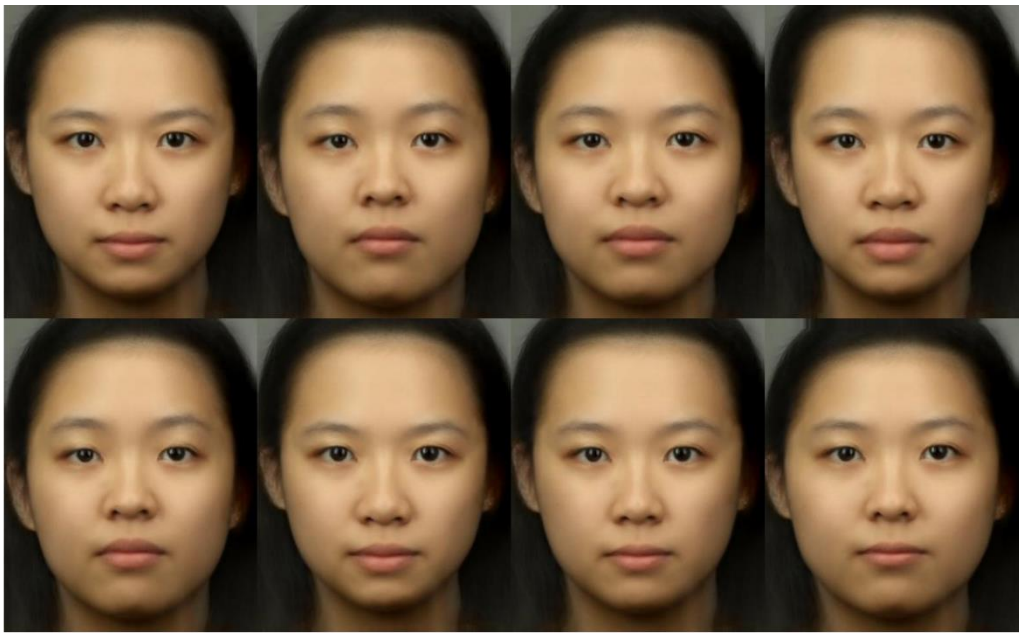

BMI (inverse) | % body fat (normalised) | BP factor | Rated health

Panel 6: Asian male

+1 sd

-1 sd

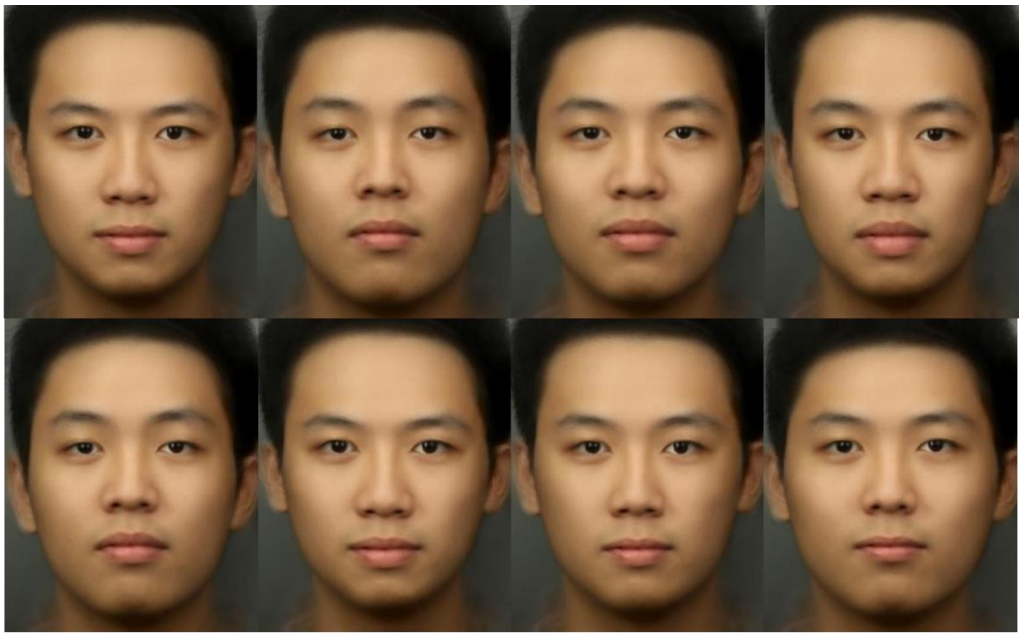

BMI (inverse) | % body fat (normalised) | BP factor | Rated health
